# Response “Commentary: a comparative study on closed reduction vs. open reduction: techniques in the surgical treatment of rotated lateral condyle fractures of the distal humerus in children”

**DOI:** 10.3389/fped.2023.1191933

**Published:** 2023-09-04

**Authors:** Liuqi Weng, Yujiang Cao, Ge Zhang, Hai Zhou, Xing Liu, Yuan Zhang

**Affiliations:** ^1^ Department of Orthopaedics, Children’s Hospital of Chongqing Medical University, National Clinical Research Center for Child Health and Disorders, Ministry of Education Key Laboratory of Child Development and Disorders, Chongqing, China; ^2^ Chongqing Key Laboratory of Pediatrics, Chongqing, China

**Keywords:** lateral condyle fractures, humerus, CRPP, ORPP, children

**A Commentary on**
A comparative study on closed reduction vs. open reduction techniques in the surgical treatment of rotated lateral condyle fractures of the distal humerus in children By Weng L, Cao Y, Zhang G, Zhou H, Liu X, Zhang Y. (2022). Front Pediatr. 11:1191933. doi: 10.3389/fped.2023.1191933

We appreciate the interests and comments (1) from Rehm et al. regarding our study entitled “A comparative study on closed reduction vs. open reduction: Techniques in the surgical treatment of rotated lateral condyle fractures of the distal humerus in children” (2) published in Frontiers in Pediatrics.

Regarding the comments on the limitations of the Song classification. To the best of our knowledge, the Song classification is a comprehensive grading system that remains widely used for lateral condyle fractures (LCFs) (3). A study from Ramo et al. (4) validated the Song's classification with high interobserver and intraobserver reliability. They also concluded that this classification improves on existing classification systems by better distinguishing fractures at risk for failure of nonoperative treatment and guiding treatment outcomes. Recently, Pressmar et al. (5) also reported that Song's classifcation provide the best practical help to estimate stability of lateral condyle fractures.

Regarding the question on the association between Milch type I fractures and an increased open reduction and percutaneous pinning (ORPP) rate. We did not employ the Milch classification for grading the LCFs in our series because it is neither predictive of outcomes nor effective in guiding treatment choices. In addition, the reliability of the Milch classification when applied in practical settings has been questioned. Mirsky et al. (6) reported that Milch's classification has not been proven to yield any prognostic value, and fractures classified radiographically were shown to have a poor correlation to intraoperative interpretation of the Fractures. Pennington et al. (7) also demonstrated that the Milch's classification was not found to be a useful tool because of the poor interobserver and moderate intraobserver agreement. Therefore, we did not use the Milch's classification as the deviations may arise from its use in further analysis. Notably, confirmed cases of Milch type I fractures are uncommon. In the present study, we included 46 patients diagnosed with Song stage 5 fractures (displaced and rotated LCFs), whereas we were not able to identify any patient with a Milch type I fracture on preoperative radiographs. Moreover, for the patients in our study who underwent open procedures, all LCFs were intraoperatively identified using direct visualization as Milch type II fractures, with fracture lines located on the medial side of the capitulotrochlear sulcus. Thus, an evaluation of the relationship between the Milch classification and the ORPP rate could not be performed in our series.

Regarding the queries on bone healing assessment. Patients with LCFs or other elbow fractures who underwent surgical treatment at our institution experienced a routine replacement of dressings and casts 1 or 2 weeks post-operatively. Thereafter, the patients were instructed to undergo clinical and radiographic evaluations at approximately 2-week intervals. Bone healing was assessed at each follow-up visit. Fracture healing was determined through a clinical examination and confirmed through the observation of a bridging callus on two radiographic views.

Regarding the rates of wound infection (superficial or deep). Surgical site infection is a common complication of surgery, and the incidence of infection after an open reduction varies among studies, ranging from 3.7% to 6.1% (8). In the present study, infections occurred only in the open reduction group; however, no statistically significant difference was detected when compared with the closed reduction group. In addition, superficial infections involve only the skin, with little or no tissue reaction, and patients usually recover well with correct and timely management. In the present study, the diagnosis of superficial infection was strictly determined and was made only if redness and swelling around the surgical incision site were observed. Collectively, superficial infections occurred in 5/36 patients who underwent open reduction. All superficial infections were managed only by daily dressing changes and topical mupirocin ointment application. All affected patients quickly healed without further use of intravenous antibiotics. However, the management of deep infection at the surgical site was indeed a challenge. In the present study, two patients who experienced deep infection were treated with intravenous antibiotics via a peripheral vein and underwent wound irrigation with saline during dressing changes. Notably, both patients recovered without further surgery. Given the limited number of patients in the present study, it is difficult to determine a reliable infection rate following surgical treatment of LCFs. However, we believe that when choosing between closed reduction percutaneous pinning (CRPP) or ORPP for the treatment of displaced and rotated LCFs, surgeons should decide based on their own therapeutic experience.

In conclusion, we indisputably support the viewpoint that CRPP is a promising technique for the treatment of displaced and rotated LCFs. However, as mentioned in our article, surgeons should be notified of the learning curve from the initiation to the skillful implementation of the CRPP technique for this type of LCF. Open reduction should always be considered as an alternative, especially when irreducible LCFs are encountered during closed procedures.
